# The American Dental Association

**Published:** 1865-08

**Authors:** 


### THE AMERICAN DENTAL ASSOCIATION.

The recent meeting of this Organization drew together a large number of the most eminent Dentists in America, and among them we were pleased to meet Dr. Taft, so long known to us through the able columns of the *Dental Register of the West*, and Dr. McQuillen, more recently associated in the editorship of the *Dental Cosmos*. The sessions were spirited, and occupied in interesting discussions on subjects of practical and scientific importance to the Dental Profession. Several lectures were delivered before the Association, and it gives us great pleasure to be able to present Dr. Brainard's address to our readers.

The hospitals and other points of interest in the city were visited, while Drs. Allport, Davis, and Miller gave very pleasant evening entertainments, and for a time the Association was most happily animated by a social, rather than professional spirit.

Had we the data from which to make it, we should be glad to give an abstract of the transactions, for we have a high regard for the Dental profession, and feel under great obligations to its members for what they have done to beautify the countenance of man, mitigate his pains, and augment the pleasures of his life.

